# Acknowledgment to the Reviewers of *Entropy* in 2022

**DOI:** 10.3390/e25020195

**Published:** 2023-01-19

**Authors:** Entropy Editorial Office

**Affiliations:** MDPI AG, St. Alban-Anlage 66, 4052 Basel, Switzerland

High-quality academic publishing is built on rigorous peer review. *Entropy* was able to uphold its high standards for published papers due to the outstanding efforts of our reviewers. Thanks to the efforts of our reviewers in 2022, the median time to first decision was 20 days and the median time to publication was 41 days. Regardless of whether the articles they examined were ultimately published, the editors would like to express their appreciation and thank the following reviewers for the time and dedication that they have shown *Entropy*:
Abah, ObinnaLima, RitaAbbasi, RezaLimbu, TejAbdalla, RifaatLin, BaihanAbdel Aleem, Shady H. E.Lin, Feng-ChengAbdel-Aty, MahmoudLin, Hsin-HonAbdelsalam, Sara I.Lin, Hsuan-YinAbdolhoseini, MahmoudLin, JasonAbdul-Sathar, Enchakudiyil IbrahimLin, Jerry Chun-WeiAbeynayake, CaniciousLin, Jun-LinAbrishambaf, RezaLin, Kuo-PingAcken, John M.Lin, MingzheAckermann, JörgLin, ShuyuAdam Erwiński, KrystianLin, Szu-YinAdamatzky, AndrewLin, ZihuaiAdami, ChristophLindsay, LucasAdhikari, SatyabrataLing, Tan HueyAdil Khan, MuhammadLipinski, DariuszAdlam, EmilyLipowski, AdamAfgan, ImranLittle, Douglas J.Afremov, LeonidLiu, ChengqingAgarwal, RaviLiu, Eric MinweiAgbehadji, Israel EdemLiu, HanAgnesi, CostantinoLiu, Hao-RanAguilar-Rodríguez, JoséLiu, HuiAhammer, HelmutLiu, JianhongAhmad, IzharLiu, JunjieAhmad, JawadLiu, LiangguiAhmad, RashidLiu, QuanhuiAhmadi, NimaLiu, RanAhmed, FaridLiu, RuipengAhmed, ShahnawazLiu, ShenghengAhmed, UmairLiu, ShuangzheAi, JunLiu, XiaokangAimone, James B.Liu, XiyaoAiriau, ChristopheLiu, YangyangAkar, NailLiu, YifanAkashi, MuhammadLiu, YongxinAkdemir, Ahmet OcakLiu, ZhungaAkhshani, AfshinLiu, ZikuiAkimoto, TakumaLivesey, Evan J.Akinyemi, LanreLiwo, AdamAkram, SheerazLiz Crespo, MaríaAktas, DjeylanLledó, CristóbalAlam, ShadabLloret, JaimeAlam, Talha MahboobLo Giudice, AntoninoAla-Nissila, TapioLo, Wai LunAlawida, MoatsumLobo, IarleyAlaya, Mokhtar Z.Lobos, Cristian Marcelo VillegasAlbahri, Ahmed ShihabLoiola, Murilo BellezoniAlbantakis, LarissaLong, GuiluAlcaine, AlejandroLongo, FrancescoAlesiani, FrancescoLoos, SarahAlessia, AlleviLoperfido, NicolaAlevtina, GostevaLopes, António M.Alexiadis, AlessioLópez Granado, Otoniel MarioAlexopoulos, KosmasLópez Pajares, DiegoAli, BabarLópez, DamiánAli, KashifLopez-Otero, PaulaAli, MehrousLópez-Yáñez, ItzamáAlizadeh Kordabad, RezaLorena, Ana CarolinaAlizadehsani, RoohallahLorencin, IvanAlkan, SoysalLorenzo Calvo, JorgeAl-Khateeb, HaiderLorenzo, SalvatoreAlkiviadis, TsimpirisLőrincz, LászlóAl-Labadi, LuaiLoskot, PavelAllen, Jeffrey B.Lotito, ValeriaAlmeida, DoraLotric, UrosAlmeida, Tiago P.Lou, YangAl-Mekhlafi, Seham MahyoubLovrić, MilivojAlshamrani, Sultan S.Lovyagin, YuriiAltintas, Azmi AliLu, HaiyuAly, Moustafa H.Lu, HeAlzubaidi, LaithLu, Qiugang (Jay)Ambrozy, TadeuszLu, YeAmeer, RashidLu, ZhiyueAmigó, José MaríaLuchinsky, AlexeyAmin, Md TanjinLucia, UmbertoAminian, GholamaliLuciano, Giuseppe GaetanoAmor, NesrineŁuczak, AleksandraAmosov, GrigoriŁuczka, JerzyAn, DongLüdge, KathyAn, DonghyeokLuef, FranzAn, ZengLughofer, EdwinAnastassopoulos, VassilisLukin, VladimirAnastopoulos, CharisŁukomska, AgnieszkaAndai, AttilaLukovszki, TamasAnđelić, NikolaLuo, ShunlongAndenoro, AnthonyLuongo, OrlandoAnderson, JohanLupu, CiprianAndersson, ClaesLupu, RaduAndrás, HorváthLushington, GerryAndreev, ValeriyLussange, JohannAndrei, MihaelaLyubushin, AlexeyAndwari, Amin MahmoudzadehMa, Der-MingAnes, VitorMa, HongyangAngelis, ConstantinosMa, HuiAngelopoulos, PanagiotisMa, JianAnsari, SamarMa, Kevin Sheng-KaiAntohi, Valentin MarianMa, SiyanAntonacci, YuriMa, XuanAntonini, MarcMa, YideAntoniou, Ioannis E.Ma, Yu-HanAono, YoshinoriMaček, NemanjaApiecionek, ŁukaszMachery, EdouardAquino, GerardoMachura, LukaszAraci, SerkanMaciel, Carlos DiasArain, SalmanMacioszek, ElżbietaAraki, YukoMaddalena, LuciaArani, Atefeh HajijamaliMadeira, LucasAraujo, FilipeMadhu, NileshAraujo, JeffersonMaffei, MariaArbi, AdnèneMagacho, Guilherme RiccioppoArcher, AntoineMagdy, NiseemArciniegas Herrera, José LuisMaglaveras, NicosArévalo, RobertoMahapatra, DurgaArgo, AntoninaMahian, OmidArgyrakis, PanosMahmoud, MohammedArgyros, Ioannis K.Mahmoudi, AminArizmendi, Constancio MiguelMahmoudi, ElhamArlazarov, Vladimir V.Mahyar, HamidrezaArmada, Manuel A.Maier, AlexanderArmaghani, Danial JahedMailthody, VikramArmoni, MichalMainardi, FrancescoArnerić, JosipMaireche, AbdelmadjidArora, SudhirMaiti, MoinakAros, RodrigoMajtey, Ana P.Arranz-Gimon, AngelMakalic, EnesArraut, IvanMakogin, VitaliiArsac, Laurent M.Makowski, MarcinArthur, RudyMakowsky, MichaelAruchunan, ElayarajaMakris, ChristosArumí, DanielMal, ShiladityaAsad, MuhammadMalaiya, Yashwant K.Asghar, MamoonaMalek-Madani, RezaAshkenazy, YossiMalgaretti, PaoloAshutosh, ChamoliMalik, NishantAsif, RameezMalkowski, ArkadiuszAsim, MuhammadMaloney, Craig E.Asjad, MuhammadMalutan, RaulAslanov, Cyril S.Man, KaiwenÅslund, JanMan, Zhong-XiaoAsorey-Cacheda, RafaelMan’ko, MargaritaAssiotis, TheoManca, VincenzoAtaei, MehdiMancini, MarcoAtstāja, DzintraMandal, ArkajitAttallah, OmneyaMangalam, MadhurAusloos, MarcelManikandan, ‪Sreenath K.Avand, MohammadtaghiManis, GeorgeAvazzadeh, ZakiehManko, Vladimir I.Avci, MustafaManolescu, AndreiAvelino, PedroMantzaris, Alexander V.Avesani, MarcoManzano, DanielAvgeris, MariosManzardo, AlessandroAwadh, Salih MuhammadMaomi, UenoAwan, Wahaj AbbasMaragakis, MichaelAydin, MuratMarascu, ValentinaAyoub, OmranMarcantoni, IlariaAzam, Naveed AhmedMarcin Laskowski, RafałAzeroual, OtmaneMarcińczuk, MichałAziz, FurqanMarcinkowski, LeszekAzzedin, FaragMarcon, EricBabazadeh, AminMarcu, Alina-ElenaBabenko, MikhailMarefati, MohammadBabichev, SergiiMares, IleanaBabik, WieslawMarey, MohamedBabiloni, Adrián MotaMargolin, DrewBacco, DavideMarhaban, Mohammad HamiruceBäcker, VolkerMarijuan, PedroBadcock, PaulMarinello, FrancescoBae, JoonwooMarine-Roig, EstelaBaek, JangsunMarinkovic, DraganBaek, MinsooMariotti, AlessandroBagavathi, ArunkumarMarkakis, Evangelos K.Bagnato, Vanderlei SalvadorMarkantonatou, StellaBagnoli, FrancoMarkovič, ReneBahrani, SimaMarkowicz, IwonaBaig, Mirza WasifMarlow, MattBairamov, IsmihanMarmur, AbrahamBajardi, FrancescoMarsiglietti, ArnaudBajic, DraganaMartello, DanieleBajrami, XhevahirMartian, AlexandruBakaev, MaximMarticorena-Sánchez, RaúlBalabanov, TodorMartin, OsmelBalachandran, VinithaMartínez Costa, María del PilarBaladron, CarlosMartínez Torres, JavierBalandin, Dmitry V.Martinez, Josu EtxezarretaBalasis, GeorgeMartinez-Corral, RosaBalcázar, NéstorMartínez-Lillo, JoséBalcerek, MichalMartinez-Val, Jose M.Baldassano, ChrisMartini, ChiaraBalenzuela, PabloMartino, AlessioBalestra, GabriellaMartino, LucaBaležentis, TomasMartsenyuk, VasylBalicki, JerzyMartyushev, LeonidBallicchia, MauroMarzen, SarahBalthazar, José ManoelMarzi, ClaudiaBalzanella, AntonioMarzuki, IsmailBamba, KazuharuMasciari, ElioBan, MasashiMaskeliūnas, RytisBan, YueMassart, EstelleBancal, Jean-DanielMastrandrea, RossanaBand, Yehuda B.Mastriani, MarioBandura, AndriyMasud, MehediBannach, MaxMasuda, ShumpeiBannone, ElisaMatejec, VlastimilBanterle, FrancescoMatera, Juan MauricioBarakat, BaselMateus, PauloBaranović, GoranMathelin, LionelBaranovskii, Evgenii S.Matsoukas, ThemisBarbancho, AnaMatsushita, RaulBarbon Junior, SylvioMattera, RaffaeleBarde, SylvainMattioni, MattiaBarel, ItayMavrantzas, VlasioBarletta, Vita SantaMayer, ChristopherBarletti, LuigiMaynar, PabloBarltrop, NigelMayo-Iscar, AgustinBarmak, OlexanderMazzilli, DarioBarra, FelipeMazzitelli, Francisco DiegoBarragán, DanielMcKague, MatthewBarranca, Victor J.Megjhani, MuradBarreca, DavideMeher, RamakantBarros, MárciaMehnen, JornBarsi, DarioMélard, GuyBartas, MartinMelbourne, JamesBaruch, ShmuelMeleppat, Ratheesh KumarBärwolff, GünterMelgarejo, Francisco ManuelBar-Yam, YaneerMelgarejo, MiguelBasagoiti, RosaMelin, PatriciaBashar, SyedMelkikh, AlexeyBasieva, IrinaMemon, SaimBasilio, MarcioMenasalvas, ErnestinaBastarrachea-Magnani, Miguel A.Mendes, MateusBastopcu, MelihMéndez, VicençBatista, ArnaldoMenghani, JyotiBauer, DietmarMensi, WalidBautu, ElenaMenvielle, LoickBaxter, GarethMercorelli, PaoloBayat, AbolfazlMerminod, SimonBeccaro, WesleyMesa, OscarBeck, ChristianMessias, MarceloBédard, Charles AlexandreMestriner, DanieleBeer, Randall D.Metelli, Alberto MariaBeerse, MatthewMeto, AidaBehl, RamandeepMezei, MihalyBekesiene, SvajoneMicallef, NicholasBekiros, SteliosMichaels, Alan J.Belikov, AlexanderMichail, TsagrisBella, GinoMichas, GeorgiosBellantuono, LoredanaMichelitsch, ThomasBellingeri, MicheleMichienzi, AndreaBellini, FabioMiedzinska, DanutaBellomo, NicolaMihail, MeganBellos, EvangelosMihalache, Sanda FlorentinaBelonoshko, AnatolyMihaljevic, Miodrag J.Belousov, Yury M.Mikkelsen, KaareBelyaev, EvgenyMikki, SaidBen Haj Frej, MohamedMiković, Aleksandar R.Ben Hamza, AbdessamadMiladinović, AleksandarBenato, AlbertoMilichovský, MiloslavBenatti, FabioMilkowski, MarcinBen-Menahem, YemimaMillan, GinnoBennett, AshleyMilovanov, AlexanderBen-Salha, OusamaMin, ZheBentley, JoeMinea, Alina AdrianaBereyhi, AliMinello, GiorgiaBergantini, AlexandreMiralles, AndréBernal-Jaquez, RobertoMiramontes, OctavioBernardes, Nadja KolbMiranda, Jose Garcia VivasBerviller, YvesMiranda, PedroBettelheim, EldadMirkes, Evgeny M.Beyabanaki, S. Amir RezaMironowicz, PiotrBhaduri, MoinakMishra, AlokBhandari, UttamMishra, AmitabhBhatia, VimalMiśkiewicz, JanuszBhattacharyya, SharmodeepMiszczak, JarekBhuiyan, Mohammad Ruhul AminMiszczak, Jaroslaw AdamBialy, MichaelMitsukura, YasueBialynicki-Birula, IwoMittal, VikramBianchi, Michele LeonardoMłynarski, WiktorBienfait, AudreyMnasri, SamiBier, MartinMobayen, SalehBieszk-Stolorz, BeataMobilia, MirkaBilén, Sven G.Möbius, ArnulfBiró, Tamás SándorMoca, Vasile VladBirou, Iulian M. T.Modarres, RezaBishop, Alan R.Modi, TusharBishwal, JayaModos, DezsoBistrian, DianaMogilevtsev, DmitryBiswas, SoumyajyotiMohamed, HanineBittencourt, Victor A. S. V.Mohamed-Seghir, MostefaBjörn, SchenkeMohammed, Beadaa JasemBlaine, TomMohan, SenthilkumarBlanter, ElenaMohapatra, ParthajitBlasiak, PawelMohd Kasihmuddin, Mohd ShareduwanBlasone, MassimoMohl, Jonathon EdwardBłaszak, MaciejMoiseev, SergeyBlaszkiewicz, LeszekMojaveri, BashirBlecha, PetrMokaev, Timur N.Blekos, KostasMol, Jan A.Blumenfeld, RaphaelMomen, SifatBlunier, SylvainMomot, AlinaBo, StefanoMondal, ChandanaBobryshev, AlexeyMonfared, MehrdadBocharova, Irina E.Montañez, George D.Bodnar, TarasMonteiro Santos, FernandoBogdanov, AlexanderMontenegro, DavisBoj del Val, EvaMontero, MiquelBolanowski, MarekMoon, JihwanBolokhov, SergeyMoon, JongsubBoloni, LadislauMoon, Seung-HyunBombek, GorazdMoon, Todd K.Bombrun, MaximeMoore, Terrence J.Bonança, Marcus V. S.Morabito, Francesco CarloBonanno, ClaudioMoraitis, NektariosBonardi, FabienMorales, PabloBonato, LucioMorandi, OmarBondar, AlexeyMoraru, LuminiţaBondi, MarinaMorawetz, KlausBonifay, WesMore, Rishabh V.Bopp, Fritz W.Morel, BenoitBorchers, ChristineMoreno, JaimeBordel Sánchez, BorjaMoreno-Armendáriz, Marco A.Borelli, DavideMorgado, GabrielBorgnakke, ClausMorgado, Welles Antonio MartinezBoriskov, PetrMorison, GordonBorovik, IgorMorita, SatoruBorowiec, AndrzejMoritz, SteffenBorowska, MartaMorosi, SimoneBosco, StefanoMorosuk, TatianaBosko, María LauraMoskowitz, IraBossé, ÉloiMotreanu, DumitruBostrem, Irina Gennad’evnaMoulos, PanagiotisBoström, KimMousavi, Seyed BorhanBotzheim, JanosMoya-Albor, ErnestoBouchaud, Jean-PhilippeMoya-Cessa, Héctor ManuelBoudraa, Abdel-OuahabMozolewska, Magdalena A.Bouri, ElieMrsic, LeoBousoño-Calzón, CarlosMrzljak, VedranBoutry, NicolasMuczyński, AndrzejBovino, Fabio AntonioMuga, GonzaloBozhko, Yulia Yu.Muhammad, Mannan SaeedBrachet, MarcMuhammad, TaseerBrad, RemusMuhammed, Hiba Z.Bradley, Robert S.Mühlhaus, TimoBrand, Alexander S.Mukerji, PurbaBrandsema, MatthewMukhamedov, FarrukhBrătian, VasileMukhametzhanov, MaratBrauneis, AlexanderMukherjee, RajBrea, JohanniMukuta, YusukeBreard, EricMuldoon, ConorBrembs, BjörnMüller, SebastianBrenner, Joseph E.Mumolo, Enzo M.Briongos, SamiraMuncharaz, Javier OliverBritz, ThomasMuñiz, CarlosBroniatowski, MichelMunoz, AlbertBrophy, MarkMuñoz, Carlos SánchezBrosch, AnnaMuñoz, EnriqueBross, Shraga I.Murari, AndreaBruce, Scott A.Mursaleen, MuhammedBrumercik, FrantisekMurugesan, SathishkumarBrunetta, CarloMuschik, WolfgangBu, YuhengMusciotto, FedericoBubnov, AlexeyMusielak, ZdzislawBuchner, TeodorMuzirafuti, AnselmeBucki, RobertNaddeo, AdeleBudini, Adrián A.Nadeem, Muhammad FaisalBudzinski, Roberto C.Nagata, KojiBuick, JamesNagy, SzilviaBujok, PetrNai Ruscone, MartaBukhari, IhtishamNaik, Ganesh R.Bukhsh, Faiza AllahNair, Arun SukumaranBull, JamesNair, ChandraBulnes, FranciscoNajjar, LotfollahBunch, HeeyounNajnudel, JosephBunea, Alina-CristinaNakamura, AtsushiBunimovich, LeonidNakano, Eduardo YoshioBuono, FrancescoNakayama, YuBurda, ZdzislawNakhla, SamBures, MiroslavNalbantis, IoannisBurg, AndreasNam, JaewonBurgdoerfer, JoachimNam, YunyoungBusato, FilippoNamasudra, SuyelBush, JohnNambu, YasusadaBuslov, MikhailNannipieri, PietroButakova, MariaNäslund, Lars-ÅkeButler, JohnNasrabadi, Maryam TayefiButt, Saah IhsanNasreddine, KamalButtigieg, VictorNasridinov, AzizButusov, DenisNassar, MazenCabaleiro, DavidNastasi, BenedettoCaban, PawelNath, PinkuCabral, BrunoNava, AndreaCacace, FilippoNavarro, SergioCaesarendra, WahyuNayel, Hamada A.Cai, ChangxiaoNaz, SumeraCai, MingyuNeagoe, MirceaCai, NingNeagu, OlimpiaCai, SiqiNéda, ZoltánCalabrese, BarbaraNejadsattari, FarshadCalado, João M. F.Nemati, HossainCalcagnile, LucioNematzadeh, HoseinCăleanu, Cătălin DanielNemzer, Louis R.Calvo, FlorentNepomuceno, ThyagoCalvo-Zaragoza, JorgeNeshat, MehdiCalzetta, EstebanNeumann, StephanCámara, Pablo G.Newcomb, RobertCambou, BertrandNguyen, Duc-PhucCamci, UgurNguyen, HoangCampo, CelesteNguyen, Manh HungCampos Cantón, EricNguyen, Ngoc Ai ThyCampos, DanielNguyen, Toan-VanCanas Rodrigues, PauloNickerson, DavidCangul, Ismail NaciNicolae, Ileana-DianaCannarile, FrancescoNie, LaisenCano, EmilioNie, QingCao, BingyangNielsen, FrankCao, JingyuNiemczyk, JerzyCao, YuanshengNieminen, PenttiCao-García, Francisco JavierNieves, Juan LuisCapone, AlessandroNigmatullin, RaoulCaporusso, NicholasNiizato, TakayukiCaprini, LorenzoNikiforov, IgorCapucci, AlessandroNikolić, Stanko N.Căpușneanu, SorinelNikolovski, SreteCarabineiro, SóniaNiola, VincenzoCaracciolo, SergioNirmalkar, NeelkanthCaraiani, PetreNiu, MinyueCaramelo, FranciscoNiu, ZhenxingCaravelli, FrancescoNkenyereye, LionelCarbone, AnnaNobre, Fernando DantasCárdenas-López, Francisco A.Noh, WonjongCardone, FabioNoor, Noor Fadiya MohdCaridad y Ocerín, Jose MariaNordell, BoCarioli, AlessandraNovak, MartinCarl, MichaelNovikova, EvgeniaCarlo, GabrielNovotný, MartinCarrasco, MiguelNtantogian, ChristoforosCarrega, MatteoNuñez-Perez, José CruzCascone, StefanoNuno, RodriguesCastellano, Nuria NovasO’Neill, JosephCastiglione, ArcangeloO’Riordan, ColmCastorrini, AlessioO’Shaughnessy, DouglasCasula, MicheleOaksford, MikeCatalina, DobreObaida, Muath A.Cataudella, VittorioOblapenko, GeorgiiCattani, CarloObregón, OctavioCavaggioni, LucaObukhov, Artyom DmitrievichCavalcanti, Rogerio TeixeiraObukhov, YuriCechinel, CristianOchowiak, MarekCeolin, AndreaOettel, MartinCerchiari, GiovanniOh, TaekeunČerný, MichalOhshima, NaokiCerqueti, RoyOhta, RyuichiCerro, GianniOikonomou, PanagiotisCervi, LauraOjo, Joseph SundayCesmeci, SevkiOjo, Mike OluwatayoChaikalis, CostasOkabe, YutakaChakrabarti, Bikas K.Okada, IsamuChallet, DamienOkolie, JudeChallis, John H.Okopinska, AnnaChalmeta, RicardoOksengendler, BorisChamberlin, Ralph V.Olaby, OsamaChan, Joseph Chang LunOlbryś, JoannaChan, Tsz NamOleynikov, Valentin E.Chanda, PritamOlivares-Robles, Miguel AngelChandra, DaryusOneto, LucaChang, Che-ChengOnken, ArnoChang, Chia-JungOrczyk, TomaszChang, Fu-MinOrganisciak, PeterChang, Hung-FuOrlik, MarekChang, Jing-RongOrorbia, Alexander G.Chang, Shun-HsyungOrozco-Duque, AndrésChang, VictorOsenda, OmarChang, Weng-LongOsińska, MagdalenaChantasri, AreeyaOsintsev, NikitaChao, AnneOsiurak, FrançoisChao, FengqingOsman, Mohamed S.Charykov, Nikolay A.Østergaard, JanChataut, RobinOstrovskii, ValeriiChatterton, StevenOsuch, PiotrChattopadhyay, EshanOsuna, ValentinChatzidimitriou-Dreismann, Chariton ArisOświęcimka, PawełChaves, Daniel A. R.Oszust, MariuszChavoshi, Seyed HosseinOtsuka, JunChen, BoOu, CongjieChen, Chin-LingOugolnitsky, GuennadyChen, HongbinOurabah, KamelChen, HongfangOuyang, TinghuiChen, HongtianOuyang, YingkaiChen, Hung-ShingOuyang, ZhenyuChen, LuonanOveis, Amir HoseinChen, MinOvseník, ĽubošChen, Ming-ChengØygarden, MortenChen, Shin-LiangOzguner, OrhanChen, Shuo-TsungOzilgen, MustafaChen, XiaoOzkaynak, FatihChen, YangOztop, HakanChen, ZhuyunÖztürk, ŞabanChen, ZiyangPachori, Ram BilasCheng, BinPaesani, StefanoCheng, DeboPaganoni, Anna MariaCheng, Fan-ChiehPaggi, HoracioCheng, Szu-ChengPagnottoni, PaoloCheng, YangPaheding, SidikeCheong, Kang HaoPaja, WieslawCheong, Siew AnnPal, NikhilChernodub, MaximPal, Priyo ShankarChernogorskiy, SergeyPaleologu, ConstantinChertovskih, RomanPaleu, ViorelChesneau, ChristophePalmero, MikelChetverikov, AlexanderPalmieri, AriannaCheung, James Chung WaiPalombi, FilippoChiaraluce, FrancoPamucar, DraganChiatti, LeonardoPan, Jeng-ShyangChickos, James S.Pan, LimingChien, Ying-RenPan, YafengChih, MingchangPanagiotakis, CostasChilingarian, AshotPanão, Miguel R. OliveiraChiper, DoruPandey, OmkantChirco, GoffredoPani, BiswaranjanChisholm, NicholasPanigrahy, ChinmayaChitov, Gennady Y.Panja, Sumit KumarCho, Myoung WonPankowska, MalgorzataChoi, Nag-ChoulPankratova, EvgeniyaChoi, SungyongPap, EndreChoi, WooyeolPapacharalampous, GeorgiaChoroszucho, AgnieszkaPapadakos, PanagiotisChotorlishvili, LevanPapadimitriou, ConstantinosChoudhary, GauravPapalexiou, Simon MichaelChoudhury, TonmoyPapenfuss, ChristinaChowdhury, SutirthaPapkova, Irina V.Chrigui, MouldiPapuzzo, GiuseppeChristov, Ivan P.Parah, ShabirChrysoula, VlachouParchin, Naser OjaroudiChu, Shu-ChuanPardo, LeandroChukhrova, NataliyaParisi, DanieleChun, JunhoParisio, FernandoChung, KyungyongPark, Ji HwanChung, Moo K.Park, Jung-MinChung, Yong-JooPark, SangkiChvostekova, MartinaPark, SangwooCicek, SerdarPark, SunhoCieśliński, JanParpa, KoullaCilluffo, DarioParthemore, JoelCimini, GiulioPasculli, AntonioCimmelli, Vito AntonioPasha, JunayedCimpoesu, FanicaPashazadeh, AliCincio, LukaszPassian, AliCiobanu, Catalin BogdanPastor, Ángel GiménezCiobanu, GabrielPastorino, MartinaCirillo, StefanoPaternostro, MauroCiuc, MihaiPathan, Al-Sakib KhanClark, NicholasPatidar, VinodCleaver, GeraldPatil, PrabhugoudaClegg, Richard G.Patro, K. Abhimanyu KumarCodetta-Raiteri, DanielePatterson, EvanCoelho, LuisPatti, AntonellaCohen, GilPaul, Titan C.Cohen, LiorPaulson, NoahCoimbra, António PauloPaun, Viorel-PuiuCojocar, Grigoreta-SofiaPaunkovic, NikolaColak, CemilPavan, GabrieleColeman, ShirleyPavlenko, IvanColonna, GianpieroPavliuk, RomanComaron, PaoloPavlyuk, DmitryComes, Calin-AdrianPavón, DiegoConard, Natasa DjurdjevacPavone, MarioConde-Cespedes, PatriciaPawela, ŁukaszCondo, CarloPawłowski, PawełConfer, MatthewPeixoto, HugoConsolini, GiuseppePekař, LiborConstantinescu, CatalinPeker, YesemConstantinescu, NicolaePeláez-Moreno, CarmenConstantinescu, RaduPele, DanielConstantoudis, VasiliosPellicane, GiuseppeConte, CarmelaPelton, ArthurCope, Thomas Peter WilliamPeña-Castillo, LourdesCoppola, GennaroPeng, GangCorcoran, PeterPeng, JiananCordeiro, WelingtonPeng, XiCorominas-Murtra, BernatPeng, XindongCorsaro, CarmeloPengo, ThomasCorti, David S.Penna, VittorioCosacak, Mehmet IlyasPennini, FlaviaCoşkun, Mustafa CemilPenttonen, MarkkuCosta, Max Henrique MachadoPeralta, Antonio FernándezCota, WesleyPerc, MatjazCotae, PaulPérez Pineda, Ramón EnriqueCox, Christopher R.Pérez-Salinas, AdriánCrane, MartinPerfors, AndrewCremaschini, ClaudioPerić, PorinCremonini, MarcoPerivolaropoulos, LeandrosCrisan, Gloria CeraselaPerlaza, Samir M.Crisan, OvidiuPerpetuini, DavidCristea, IrinaPerumal, ArumugaCrokidakis, NunoPetegrosso, RaphaelCruciani, EmilioPeter, Adrian M.Cruz, GonçaloPéter, MukliCruz, ManuelPeterson, Kara J.Cruz-Aceves, IvanPetiziol, FrancescoCsató, LehelPétritis, DimitriCubero, Ryan JohnPetritoli, EnricoCumming, DouglasPetrov, AlbertCuriac, Daniel-IoanPetrov, Alexander N.Cyrus, VáclavPetrov, Arthur I.Cysouw, MichaelPetrović, MilicaCzachor, MarekPetzoldt, AlbrechtCzarnowski, IreneuszPeukert, BerndCzerwinski, ArturPfost, HeinerD’Abramo, GermanoPham, Quoc BaoD’Agosta, RobertoPham, ThongD’Ariano, GiacomoPham, Viet-ThanhD’Oliveira, Rafael G. L.Piangerelli, Marcoda Conceição Costa, MariaPiantadosi, Steven T.da Mota Vilela, André LuisPiao, Guanyuda Silva Filho, Olavo LeopoldinoPiasini, Eugenioda Silva, José MachadoPichardo, Eduardoda Silva, SergioPiechocki, Włodzimierzda Silva, Sérgio LuizPieczynski, WojciechDaanial Khan, YaserPietrowicz, SławomirDahash, AbdulrahmanPigeon, StevenDai, XiaoxiaPijarski, PawełDairi, AbdelkaderPileggi, Salvatore F.Dajka, JerzyPilmane, MāraDalarsson, MarianaPilotelli, MariagraziaDamaševičius, RobertasPinčák, RichardDamer, BrucePinchetti, ValerioDamián-Ascencio, César EduardoPineda, MiguelDamjanović, BoškoPinho, Armando J.D'Andrea, AntonioPini, DavideDaneshazarian, RezaPinoli, PietroDaneshkhah, AlirezaPintea, Camelia M.Dang, Duc Ngoc MinhPintelas, PanagiotisDang, Thanh DucPîrjan, AlexandruDaniels, BryanPirjol, DanDaovisan, HanvedesPirner, MarliesDarmon, DavidPiroddi, LucaDas, DebarshiPirogov, SergeyDas, DeepyamanPistone, GiovanniDas, HimansuPiteľ, JánDas, RishabhPlakandaras, VasiliosDas, ShibanandaPlastino, Ángel RicardoDaub, ChristopherPlastino, AngeloDauhoo, MuhammadPlata, Carlos A.Daunys, GintautasPlechawska-Wójcik, MałgorzataDautriche, IsabellePleimling, MichelDavelaar, Eddy J.Ploskas, NikolaosDavid, Sergio A.Plotnitsky, ArkadyDavies, Paul C. W.Poggie, JonathanDavis, JamesPogudin, GlebDavydenko, YevhenPolańczyk, AndrzejDayananda, Surabhi GarudangiriPoletti, DariodC Rubin, Sergio S.Poměnková, Jitkade Alencar Filho, Geová MacielPonomaryov, Volodymyrde Alencar, Marcelo SampaioPons, Miquelde Almeida, Norton GomesPop, Nicolaede Brevern, Alexandre G.Poparić, GoranDe Conti Teixeira Costa, GustavoPopescu, Paulde Corato, MarcoPopkov, Yuri S.de Faria, José Geraldo PeixotoPorta, Albertode Jesus León-Montiel, RobertoPortillo, Davidde la Fuente-Mella, HannsPosadas, Antonio M.de la Peña, LuisPoskrobko, Sławomirde la Sen, ManuelPotel, Gregoryde Lima Bernardo, BertioPotika, Katerinade Michele, CarloPotirakis, Steliosde Morais, Gomes RafaelPotužák, Tomášde Oliveira Júnior, SilvioPoulose, Alwinde Oliveira, Marcelo MartinsPourabdoli, Mehdide Paula Neto, Fernando MacianoPourkarimi, Mohammad Rezade Sanctis, Angela AnnaPovstenko, Yuriyde Santiago-Perez, J. JesúsPowroznik, Pawelde Sousa Lima, Francisco W.Prabakaran, Vivekhde Tommasi, LucianoPradana, Muhamad Hilmil Muchtar AdityaDeaconu, AdrianPrado-Velasco, ManuelDeaconu, Sorin IoanPrakash, ChanderDębowski, ŁukaszPrangemeier, TimDechoum, KaledPrasannakumara, Ballajja C.Decu, SimonaPrata, João NunoDedu, SilviaPrayudi, YudiDegollado, Juan CarlosPreda, VasileDehak, RedaPregowska, Agnieszkadel Barrio Castro, TomásPrelipceanu, MariusDelcea, CameliaPremaratne, PrashanDelić, VladoPremkumar, ManoharanDelsole, TimothyPrentner, RobertDelwar, Tahesin SamiraPrestipino, SantiDemianski, MarekPrice, Christopher JohnDemidova, LiliyaPrieto Guerrero, AlfonsoDemirel, YasarPrieto, Francisco UreñaDeng, ShangkunPritykin, DmitryDeng, ShuyanProkeš, AlešDenti, EnricoProkopidis, KonstantinosDepken, CraigPryss, RüdigerDeppman, AirtonPrzybyła-Kasperek, MałgorzataDeryabin, MaximPrzystupa, KrzysztofDeufemia, VincenzoPsilovikos, ArisDewi, ChristinePtaszyński, KrzysztofDi Candia, RobertoPu, Yu-ChiDi Capua, RobertoPuchinger, MarkusDi Caro, LuigiPuebla, RicardoDi Cosmo, FabioPuechmorel, StephaneDi Crescenzo, AntonioPulido, BelarminoDi Prisco, AliciaPunn, Narinder SinghDi Renzo, FrancescoQamar, FaizanDialektopoulos, KonstantinosQi, FengDiamantopoulou, VasilikiQi, GuanqiuDias, TiagoQian, Wei-LiangDiatta, AndréQin, MinghaiDiaz, Rocio GonzalezQin, WeiDickert, Franz L.Quach, JamesDief, TarekQuapp, WolfgangDieks, DennisRacz, Frigyes SamuelDíez-González, JavierRadac, Mircea-BogdanDijk, Tom VanRadic, DankoDimililer, KamilRadivilova, TamaraDimitriadis, Panayiotis G.Rae, AlastairDimitriou, DimitriosRafatirad, SetarehDimitrov, AlexanderRafiqul Islam, Md.Dimitrov, Vassil S.Ragulskis, MinvydasDin, AnwarudRagusa, Maria AlessandraDing, Jian-JiunRahbari, DadmehrDing, JupengRahman, Md MamunurDing, NiRahman, MuhibUrDionísio, AndreiaRai, Dibya PrakashDioşan, LauraRailean-Plugaru, VioricaDiraco, GiovanniRaimondi, GianfrancoDjorwé, PhilippeRaja, KalpanaDo, Xuan PhuRajaguru, GulasekaranDobeš, JosefRajmic, PavelDobre, Robert AlexandruRajput, DharmendraDobreva, AntoanetaRak, RafałDodig-Crnkovic, GordanaRam, MangeyDodonov, ViktorRaman, RamakrishnanDohnal, FadiRamesh Babu, AshwinDolatabadi, NaderRamírez Pacheco, Julio CésarDoll, KlausRamirez, Pedro E.Dolnik, BystrikRamirez-Reyes, AbdielDomcke, WolfgangRamirez-Rojas, AlejandroDominguez, DavidRamm, BrentynDoncel, JosuRamón-Cardona, JoséDong, GaogaoRamos-Vidal, IgnacioDong, ZuominRamtin, Amir RezaDonner, ReikRana, PratipDonovan, RichardRangarajan, Shriram S.Donskoy, IgorRangel-Magdaleno, José de JesúsDopazo, CesarRanjan, Premdos Reis, GonçaloRansing, Rajeshdos Santos Lim, LeonardoRapisarda, Andreados Santos, MarcosRappel, HusseinDospinescu, NicoletaRasappan, SureshDouven, IgorRashad, Ahmed M.Dover, YanivRashidi, JouanDowlatshahi, Mohammad BagherRashkovskiy, Sergey A.Doyle, JosephRasouli, SeyedDraganić, AndjelaRast, PhilippeDragoi, MarianRastelli, GianlucaDragoș Daniel, ȚarălungăRath, PratikDresp-Langley, BirgittaRatnasingam, SuthakaranDrewniak, JózefRauf, Hafiz TayyabDrezet, AurélienRavignani, AndreaDrezewski, RafalRavindra, Nuggehalli M.Drivas, Theodore D.Ray, ShannonDroge, GregRaz, OrenDrozdz, StanislawRaza, ImranDruzhinin, OlegRazavi, MohsenDu, JunRazeghi, BehroozDu, Wei-ShihReboiro, MartaDu, ZhidongRebolledo, RolandoDuan, YifeiRebufello, EnricoDuarte, IsabelReddy, Patakota SudarsanaDudczyk, JanuszRehman, AbdulDulebenets, MaximReisslein, MartinDulf, Eva H.Ren, JamesDumitru, Cristian-DragoşRen, LuDunaev, Anatoliy M.Ren, YongDuncan, EarlRenda, MarioDunkel, JörnRenedo, Carlos J.Dupraz, ElsaRenigier-Bilozor, MalgorzataDurai Raj Vincent, P. M.Renzi, CristinaDuraj, AgnieszkaResende, PedroDurán Díaz, IvánRestocchi, ValerioĎurčanský, PeterRevetria, RobertoDwivedi, Ashutosh DharRezazadeh, HadiDyakin, Victor V.Rezvani, MohsenDzemyda, GintautasRhi, Seok-HoDzhafarov, EhtibarRiaboff, LucileDzhunushaliev, VladimirRiad, KhaledDziedzic, AndrzejRiascos, Alejandro PérezDziembowski, AdrianRibeiro Gallo, Waldyr LuizDzurenda, PetrRibeiro, Maria Isabel BarreiroEdelen, JonathanRibeiro, TiagoEdet, CollinsRicci, LeonardoEdward, KrzyżakRicciardi Celsi, LorenzoEgger, JanRice, JohnEgiazarian, KarenRichmond, Christ D.Egle, Tomasi-GustafssonRichmond, PeterEgorov, VladimirRichter, Wolf-DieterEiras Fernández, JesúsRida, ImadEjdys, JoannaRing, PeterEkici, SelçukRios-Figueroa, Homero VladimirEkmekci, IsmailRioul, OlivierEl Assad, SafwanRipoli, AndreaEl Hasadi, YousefRivero-Angeles, Mario E.El Oualkadi, AhmedRizvi, Syed Tahir HussainElaziz, Mohamed AbdRizzi, MariaElbaşı, ErsinRobert, PhilippeEleschová, ŽanetaRoberts, GarethEleuch, HichemRobitzsch, AlexanderElgendy, IbrahimRobles, Ramiro SamanoEl-khatib, YoussefRoccetti, MarcoElkina, AnnaRodari, GiovanniEl-Morshedy, MahmoudRodin, Andrei S.Elmouki, IliasRodkin, MikhailEl-Shafie, MostafaRodrigues, AlexandreEl-Zahar, EssamRodrigues, DiogoEmekwuru, NwabuezeRodriguez, Denis DelisleEmura, TakeshiRodriguez, NibaldoEntezami, AlirezaRodriguez-Tello, EduardoErcan, FurkanRogerio Pinheiro, PlácidoErcegović Ražić, SanjaRogowski, KrzysztofErdebili, BabekRoh, HeejunEscudero, JavierRohan, AliEsquivel, Rodolfo O.Roig, Ignacio BoschEstévez, Pilar GarciaRoka, RastislavEvans, MichaelRokicki, TomaszEvans, Peter W.Roli, AndreaEwees, Ahmed A.Romatschke, PaulEzra, Gregory S.Romero-Rochin, VictorFabian, Heidrich-MeisnerRonco, MicheleFabián, ZdeněkRong, JiaFachechi, AlbertoRonzhina, MarinaFaísca, Patrícia F. N.Rosas, FernandoFakhimi, SiavashRoscia, MariacristinaFakotakis, NikosRosinová, DanicaFalasco, GianmariaRosiński, AdamFalcão, AndreRossi, GiancarloFalkowicz, KatarzynaRossi, RiccardoFalkowski-Gilski, PrzemyslawRostami, MehrdadFan, WeiRostami, MohammadFan, ZhengpinRoszkowska, EwaFang, HuiRotariu, CristianFang, ShuiliangRoterman, IrenaFaouzi, JohannRotter, PawelFarajpour, AliRotundo, GiuliaFarantos, Stavros C.Rovenchak, AndrijFarias, Francisco Werley CiprianoRoversi, José A.Farid, Hafiz Muhammad AtharRoy, AnkitFarina, LorenzoRoy, DibyenduFarnsworth, Keith D.Roy, PinakiFavretti, MarcoRoy, SurajitFazio, PeppinoRozas-Fernandez, AlbertoFedorov, Ruslan V.Ruan, SuFeichtinger, Hans GeorgRuan, ZhongyuanFeketa, PetroRubega, MariaFekih, AfefRubi, Miguel J.Feldhoff, ArminRubinov, MikailFeleaga, LilianaRubio Alonso, HiginioFelice, DomenicoRubio-Loyola, JavierFelici-Castell, SantiagoRuchay, AlexeyFellerman, HaroldRumyantsev, KonstantinFelseghi, Raluca AndreeaRuocco, GianpaoloFeng, KeRuslans, SmiginsFeng, LonglongRusso, PaoloFeng, MinyuRussomanno, AngeloFerilli, StefanoRyabko, BorisFernandes, Carlos M.Rybin, PavelFernandez Oro, Jesus ManuelRybin, VyacheslavFernandez, Luis ManuelRydzewski, JakubFernandez-Veiga, ManuelRyumin, DmitryFernando, Molina-GranjaSaad, Khaled MohammedFeroze, NavidSaadane, RachidFerrario, MauroSaadat, Mohammad HosseinFerraro, DarioSaadati, RezaFerrazani Mattos, Diogo MenezesSaavedra, JorgeFerreira, Antonio LuisSaber, Wardiya AfsharFerreira, ArturSabido, MiguelFerreira, PauloSabin, CarlosFerreira, Rui M. L.Sabry, AmrFetaya, EthanSabuncu, MetinFiala, PavelSacco, Pier LuigiFidaleo, FrancescoSachlas, AthanasiosFidanova, StefkaSadaati, RezaFields, ChrisSadun, Lorenzo A.Figà-Talamanca, GiannaSaeed, Syed TauseefFike, MatejSáez, José A.Filchenkov, AndreySafarpour, HamedFilippov, AndreySafdari Shadloo, MostafaFilippov, SergeyŠafránek, DominikFino, Anna MariaSafron, AdamFishman, DmytroSagar, Robin P.Fiumara, GiacomoSaghiri, Ali MohammadFiurasek, JaromirSahu, Aditya KumarFivos, PapadimitriouSahu, BinodFleischmann, RagnarSaida, HiromiFliss, Jackson R.Saito, ShimpeiFlora, Bruno Felice FilippoSajjadi, HasanFlores, Juan CésarSakalauskas, VirgilijusFlores-Márquez, Elsa LeticiaSakon, TakuoFlori, AndreaSalahdine, FatimaFlorio, GiuseppeSalamanos, NikosFodorean, DanielSalamon, PeterFolifack Signing, Vitrice RubenSalas Piñón, JuliánForeman, MatthewSalazar, AddissonForestier, GermainSalazar-Colores, SebastiánFortuna, LuigiSaleem, SalmanForuhandeh, MahsaSalehi, Mohammad JavadFoschini, LuigiSalini, SilviaFouda, Jean Sire Armand EyebeSalmane, PascalFountoulakis, EmmanouilSaltı, MustafaFourer, DominiqueSaltykova, OlgaFournier, SébastienSalvadori, SimoneFox, ColinSamanta, DebabrataFrąckiewicz, PiotrSamil Demirkol, AhmetFragos, VassiliosSamson, Edward CarloFrancavilla, CristianSanches, Danilo SipoliFranceschetti, LorenzoSánchez Lasheras, FernandoFranceschini, AndreaSanchez Ramos, JoseFranci, Alessio FranciSánchez-Pérez, Juan FranciscoFrattolillo, FrancoSanchez-Soto, LuisFrigurǎ-Iliasa, Flaviu MihaiSánchez-Soto, Luis L.Friston, KarlSands, DavidFritschek, RickSanei, SaeidFrolov, NikitaSanjuán, Eva LópezFrydryszak, AndrzejSankaranarayanan, BathrinathFu, ChongSantamaria-Holek, IvanFu, GangSantamato, AlbertoFu, Tak-chungSantiago-Rodríguez, EfraínFu, XiangSantoro, AdolfoFu, Yuejiao (Cindy)Santos, AndrésFuh, Chiou-ShannSantos, RodrigoFukui, KosukeSanz Ortiz, Ángel SantiagoFülöp, BazsóSanz, Ángel S.Fung, Tsz ChaiSanz, RuyFurmańczyk, KonradSapa, LucjanFurui, SadatakaSarabia, José MaríaFuruichi, ShigeruSaracco, FabioFusco, GiuseppeSaranti, AnnaFushing, HsiehSarasa-Cabezuelo, AntonioGabriela, MirceaSarjas, AndrejGadekallu, Thippa ReddySarkar, AritraGaitán-Angulo, MercedesSarkar, DebajyotiGalati, GaspareSarkar, SobhanGaleazzi, AlessandroSarker, PranabGalley, Thomas D.Sarlis, NicholasGallos, LazarosSarmadi, HassanGalofaro, FrancescoSarris, Ioannis E.Galyaev, AndreySarwar, MusavarahGambelli, AlbertoSasai, KazutoGammaitoni, LucaSasaki, YuyaGangopadhyay, AryyaSason, IgalGangopadhyay, SunandanSatler, MassimoGao, CongSato, HiroyukiGao, JingenSato-Ilic, MikaGao, LiSavazzi, PietroGao, LiangSavoiu, GheorgheGao, WeidongSawerwain, MarekGao, XiangyunSawicki, JanGao, ZhiweiSaxena, SahajGarcía Hernández, José JuanSazim, SheikhGarcía Suárez, Víctor ManuelSbert, MateuGarcía, ÁlvaroScalera, LorenzoGarcía, Jesus EnriqueScarfone, Antonio M.García-Frias, JavierScelles, NicolasGarcía-García, Leonardo AzaelSchäfer, BenjaminGarcía-Mateos, GinésSchatten, MarkusGarcía-Medina, AndrésSchelenz, RalfGarcia-Moreno, AdrianSchilcher, UdoGarcia-Pinedaa, MiguelSchinckus, ChristopheGarcin, MatthieuSchindler, JosephGarda, BartłomiejSchlindwein, Fernando SoaresGarg, PrakharSchlitter, JürgenGasaneo, GustavoSchmeidel, EwaGasimov, Yusif S.Schmidt, Jan H.Gast, VolkerSchmitz, RolandGatto, Bernardo B.Schmoch, UlrichGaur, ManasSchoenberg, FredericGauthier, Daniel J.Schroeder, Marcin J.Gavrilas, MihaiSchuler, AlejandroGavrilova, MarinaSchumann, AndrewGayialis, Sotiris P.Schuster, SebastianGe, HaoSchwenker, FriedhelmGe, WenjunSciacovelli, LucaGeelan, DavidSciandra, MariangelaGell, David A.Scott, Tony C.Geng, TianyuScotto, ManuelGenoni, Marco G.Sebastián, AndrésGeorgiades, PantelisSeck-Tuoh-Mora, Juan C.Georgiev, DankoSedki, MohammedGeorgiou, ChristosSeely, Andrew J. E.Georgoudas, IoakeimSegell, GlenGeradts, ZenoŠegota, Sandi BaressiGerman-Sallo, ZoltanSegundo-Ortin, MiguelGershenson, CarlosSeibold, GoetzGershman, SamuelSeijas-Macias, AntonioGhaffari-Hadigheh, AlirezaSeixas, JoseGharehdash, SabaSekhar, JainageshGhauri, Sajjad A.Selimefendigil, FatihGhayoula, RidhaSellitto, MiguelGheorghe, GrigorasSellountos, EuripidesGhimire, DeepakSemenov, Vladimir I.Ghiu, IuliaSemlitsch, BernhardGhoniemy, SamySeo, Hun-ChulGhorbani, ModjtabaSepúlveda, DanielGhosh, AnimikŞerban, GheorgheGhosh, AsheshSerdaroğlu, GoncagülGhosh, BikramadityaSergi, AlessandroGhosh, DebashisSerikov, ArkadyGhosh, IndranilSerra, PauloGiannoccaro, Nicola IvanSerrano Chica, Jose-MariaGibson, Jerry D.Serra-Ruiz, JordiGiełczyk, AgataServi, MichaelaGielen, SteffenSesma-Sara, MikelGigante, GabriellaSessa, SalvatoreGilev, BogdanSestak, JaroslavGil-Villegas, AlejandroSevaux, MarcGiordano, StefanoSevilla, Francisco J.Giorgi, Gian LucaShabir, MuhammadGiraldo, RamónShaginyan, Vasily R.Girault, Jean-MarcShah, PuravGirovský, PeterShahriyari, LeiliGlavatskiy, KirillShaik, SasonGléria, Iram MarceloShakeriaski, FarshadGlösekötter, PeterShamrat, F. M. Javed MehediGluzman, SimonShao, KeGniadecki, RobertShao, SicongGo, Tae-SikSharma, AnkitGodinho, Jose R. A.Sharma, SancharGodoy, DanielaSharov, German SergeevichGoga, NicolaeSharp, MichaelGogola, MariánShaukat, KamranGoi, Bok-MinSheikh, Nadeem AhmedGołdasz, AndrzejSheikholeslami, MohsenGoldstein, MosheSheka, ElenaGolmankhaneh, Alireza KhaliliShendryk, ViraGoltsev, AlexanderSheremet, MikhailGomes, CláudioSherwin, William B.Gomes, RicardoSheu, Jeng-ShinGómez, Héctor W.Shi, ChangchengGoncharov, Anton A.Shi, HongliangGong, YunguiShi, WenbinGonzalez-Ayala, JulianShi, XiaopingGonzález-de-la-Rosa, Juan-JoséShi, YanlinGonzalez-Espinoza, ManuelShih, ChihhsiongGonzalez-Parra, GilbertoShimotomai, MichioGonzález-Pérez, Pedro PabloShin, JunbumGonzález-Rodelas, PedroShinohara, SusumuGoodwell, AllisonShioda, ShigeoGórecki, TomaszShiue, Muh-TianGorgan, DorianShlesinger, Michael F.Górniewicz, LechShleymovich, MichailGorshenin, Andrey K.Shokrollahi, FoadGosak, MarkoShu, MinGoschin, ZiziShuai, JianweiGottschalk, SylviaShudo, AkiraGouba, LaureShukla, RohitGoupil, AlbanSicuro, GabrieleGouveia, SoniaSidler, DominikGrabinski, MichaelSidorov, Sergei PetrovichGrabowska, KarolinaSikora, MałgorzataGradojevic, NikolaSilagadze, Zurab KarlovichGrande-de-Prado, MarioSilva, AdãoGreen, DaleSilva, ArlindoGreen, David GeoffreySilva, Luiz Eduardo VirgilioGreenberg, ShlomoSilva, NunoGreenewald, Kristjan H.Silva, Rui VascoGriffiths, BobSimate, Isaac N.Griffiths, Ryan-RhysSimões, PaulaGrigorescu, AdrianaSinger, GonenGrimm, VolkerSingh, AmarGrisolia, GiuliaSingh, DilbagGrmela, MiroslavSingh, NikitaGrochla, KrzysztofSingh, PrashantGromov, Vasilii A.Singh, ShellyGrøn, ØyvindSingleton, DouglasGroniewsky, AxelSiniscalchi, Sabato MarcoGrosch, TheodoreSioris, Christopher E.Grossman, EitanSiqueira, Hugo ValadaresGrosu, LaviniaSiqueiros, Jesús M.Gschwandtner, UteSirbu, Ioana-GabrielaGu, BingSitar-Tăut, Dan-AndreiGu, HengyuSitaula, ChiranjibiGuarda, TeresaSivasankaran, SubbarayanGuardabascio, BarbaraSkarbek, WładysławGubiec, TomaszSkokowski, PawelGuccione, SalvatoreSkoulikaris, CharalamposGudelj, AnitaSkrobek, DorianGuerrero-Sanchez, Maria EusebiaSładkowski, JanGuida, AlessandroSlimani, YahyaGuimarães, MarceloŚliwa, IzabelaGumulya, MonicaSlobodov, AlexanderGumuscu, BurcuSmilansky, UzyGunes, UmitSmirne, AndreaGuo, Chao-YuSmirnov, Nickolay N.Guo, FuchunSmotrovs, JurisGuo, JunchengSobota, TomaszGuo, LongxiangSoco, EleonoraGuo, LuluSodhro, Ali HassanGuo, Si-KaoSofronov, GeorgyGuo, ZhongyinSohail, MuhammadGupta, DeepakSohani, AliGupta, NishuSolanki, SourabhGutkin, BorisSole, PatrickGwosch, ThomasSoleymani, MohammadGyulchev, GalinSoloviev, Vladimir O.Ha, DavidSolovyov, AndreyHadar, OferSomvanshi, Pramod R.Haddag, BadisSong, BofanHadfield, StuartSong, JingweiHadi, Muhammad UsmanSong, QinghaiHafeez, AzeemSong, TianqiHaglund, JesperSonkusare, ReshmaHaider, JunaidSonobe, ReiHailu, GetuSorensen, JeffreyHajduk, ZbigniewŠorli, AmritHalanay, AndreiSosnowski, MarcinHamamci, Serdar EthemSosnowski, Zenon A.Hamed, AhmedSottocornola, GabrieleHamid, YasirSoumplis, PolyzoisHammad, Mohamed AdelSouravlas, StavrosHamza, BenSouza, Richard DemoHamza, RafikSowa, Jakub K.Han, BinSpanbauer, CharlesHan, JianingSparavigna, Amelia CarolinaHan, QinkaiSpiechowicz, JakubHanczyc, MartinSpindler, MartinHanias, MichaelSpivey, Michael J.Hankin, RobinSquartini, TizianoHannig, JenniferSridhar, KaushikHansen, Lee D.Srinivasulu, AvireniHao, FeiSrivastav, Akhil KumarHao, YajiangStai, EleniHarding, BrendanStamou, AdamantiaHarezlak, KatarzynaStanescu, Nicolae-DoruHarremoës, PeterStankeviciene, JelenaHarrison, WillieStankovic, RadomirHaruna, TaichiStavrinos, PanayiotisHasan, NajamStavrou, Photios A.Hasimoto-Beltran, RogelioStawowy, MarekHassan, AhmadSteel, MikeHassanabadi, HassanStefanatos, DionisisHassanpouryouzband, AliakbarStefaniak, PawełHategan, Camelia DanielaStefanou, PavlosHatsuda, YasuyukiSteinberg, SimonHauke, JanStein-O’Brien, GenevieveHaven, EmmanuelStella, LorenzoHavig, PaulStella, MassimoHawkins, RaymondStepanov, Sergey P.Hayat, UmarStephanou, PavlosHaydn, NicolaiStępień, KrzysztofHe, Jianxun (Jennifer)Stern, JulioHe, YangboStern, SebastianHe, YugangŠtikonas, ArtūrasHe, ZhiqiStipčević, MarioHead-Marsden, KadeStoica, FlorinHegedűs, FerencStoica, Ovidiu CristinelHegedus, PeterStoica, SabinHeindorf, StefanStoupos, NikolaosHejna, BohdanStoyanov, BorislavHeld, AaronStránský, PavelHeld, ChristophStrauss, AlfredHeliodoro, PaulaStrawn, NateHentschel, MartinaStreckiene, GiedreHerber, Daniel R.Strömbom, DanielHerlau, TueStrozzi, FernandaHerlem, GuillaumeStrušnik, DušanHerman, EmiliaStrzałka, DominikHernández, ÁngelaŠtula, MajaHernández-Fernández, AntoniStutzer, MichaelHernes, MarcinSu, GuoxinHerrera Corral, Gerardo A.Su, RuoyuHerrmann-Pillath, CarstenSuárez Gómez, Sergio LuisHerzog, BodoSuchecki, KrzysztofHess, KarlSuciu, AlinHess, SibylleSudbery, AnthonyHeszberger, ZalánSugano, MasashiHetreux, RaphaeleSulis, WilliamHickinbotham, SimonSummers, Richard L.Hidalgo, ArturoSun, Guo-HuaHidalgo-Herrero, MercedesSun, JieHigson, EdwardSun, JunHildebrandt, AndreSun, KeHills, ThomasSun, Li-HsienHilt, AttilaSun, RuoxiHingerl, KurtSun, TongjunHinrichs, HermannSun, ZhaoxiHinsch, MartinSundar, BhuvaneshHirche, ChristophSung, Sheng-FengHirose, YuichiSung, Tae-EungHo, Eric S.Suprano, AlessiaHo, JosephSupriya, SupriyaHo, Le BinSuravajhala, PrashanthHo, Steven Sai HangSuraweera, Himal A.Hoang, Tiep M.Surian, DidiHoang, Trong-ThucSuris, YuriHodge, VictoriaSuss, Matthew E.Hofer, MatthiasSutherland, KennethHoffmann, Karl HeinzSuzić, SinišaHoffmann, MarkoSuzuki, JunHolik, FedericoSuzuki, ReijiHolmes, DawnSvanadze, Maia M.Holmes, JeremySvozil, KarlHolovatch, YurijŚwiątek, JerzyHolzinger, AndreasŚwiercz, MirosławHong, Chang-YuŚwietlicka, AleksandraHong, PengyuSylvia IV, J. J.Hong, SungbumSyriopoulos, KonstantinosHong, Wei-ChiangSyropoulos, ApostolosHong, YingSzczepaniuk, HubertHorák, JakubSzczepanski, JanuszHorn, BartSzczęsna, AgnieszkaHorng, Ji-HweiSzkaliczki, TiborHornung, RomanSzpica, DariuszHorri, NadjimSzwarc, KrzysztofHorswill, Craig A.Szymanski, Boleslaw K.Horvatić, DavorTaasti, Vicki TrierHorwitz, LawrenceTaghavifar, HadiHossain, MoyazzemTagliaferri, RobertoHosseini, Seyed AliTaha, Ibrahim B. M.Hosseinichimeh, NiyoushaTaj, DavidHosseinzadeh, KhashayarTakayasu, HidekiHosseinzadeh, MehdiTalanov, MaxHost-Madsen, AndersTalebi, Sayed PouriaHotta, Luiz K.Talebizadeh Sardari, PouyanHou, WeizhenTaler, DawidHoussein, EssamTama, Bayu AdhiHovd, Martha NorbergTamascelli, DarioHoyos Velasco, FredyTamm, Mikhail V.Hsiao, Kai LongTamulevičius, GintautasHsiao, Sung-JungTan, CaoHsiao, Tzu-ChienTan, HongboHsieh, Fu-ShiungTanveer, SheikHsieh, Ping-ChengTapia-Rojo, RafaelHsu, Chao-MingTapias, DiegoHsu, Li-YiTarafdar, SouravHsu, Ming-FuTarasov, Vasily E.Hsu, Ming-HungTarassyev, AlexanderHsu, Terng-YinTartaglia, AngeloHu, GangTartaglione, MichelaHu, HaoTashman, Deemah H.Hu, LiangxingTauveron, NicolasHu, TingTchemisova, TatianaHu, Zhan-YiTcholtchev, NikolayHu, ZhongxuTchórzewska-Cieślak, BarbaraHua, ChengyunTegos, AristotelesHuang, ChaomingTeiko, HeinosaariHuang, GordonTeixeira Alves, DaniloHuang, Guan-ShiengTeixeira, João Paulo RamosHuang, Hsin-HsiungTek, SuleymanHuang, Jheng-JiaTelcs, AndrásHuang, Jih-JengTelesca, LucianoHuang, ShaoguangTenBarge, JasonHuang, TaoTeng, Huei-WenHuang, WeiminTeodoro, Maria FilomenaHuang, XianzhengTerna, PietroHuang, Yung-FaTerven, JuanHubka, LukášTerzyiska, MargaritaHübner, WolfgangTeslyuk, VasylHudecová, ŠárkaTessadori, JacopoHung, Jui-ChengTessarotto, MassimoHussain, MuhammadThalassinos, EleftheriosHussain, NiamatThan, Vo-VanHussain, ShujaatThingna, JuzarHussain, ZahirThomas, AndrewHutter, MarcusThomas, JohnHuyer, WaltraudThongtip, TongchanaHyun, EuginTian, ChushunIana, Vasile-GabrielTian, JingIannone, RaffaeleTierney, Nicholas J.Iantovics, Laszlo BarnaTiezzi, MatteoIasiello, MarcelloTimokhin, MaksimIbrahim, MohamedTimoney, JosephIchikawa, TsubasaTintori, AndreaIeracitano, CosimoTiomkin, StasIgasaki, TomohikoŢiplea, Ferucio LaurenţiuIghravwe, DesmondTirnakli, UgurIiboshi, HirokuniTitarev, VladimirIkegami, TakashiTkach, ItshakIlić, Velimir M.Todeschini, SaraIlieva, GalinaTodorov, ValentinIllias, Hazlee Azil BinToenjes, RalfImre, Attila R.Toffano, ZenoIngber, LesterTokuhiro, AkiraInkermann, DavidToma, AidaInoan, Daniela IoanaToman, BlazaInsausti, XabierTomasic, IvanIoannis, SarrisTomasin, StefanoIomin, AlexanderTomassini, MarcoIonac, NicoletaTomaszewski, DamianIovanella, AntonioTomczyk, KrzysztofIrshad, MuhammadToomaj, AbdolsaeedIsaic-Maniu, AlexandruToral, RaúlIsar, AurelianTorres, GermánIslam, Md TanvirulTorres, Javier MartínezIslam, MuahammadTorri, Marco Danilo ClaudioIslam, RiadulTorrieri, GiorgioIsmael, JenannToshmatov, BobirIvanescu, Andrada E.Tosic, PredragIvanescu, MirceaTosta, Thaina Aparecida AzevedoIvanis, PredragTosun, Pinar DenizIvanov, DaniilTrakadas, PanagiotisIvanov, DenisTrassinelli, MartinoIvanov, OvidiuTravieso, GonzaloIvanov, Peter AleksandrovTreesatayapun, ChidentreeIvanov, Sergei I.Triantafyllou, IoannisIvanova, MalinkaTridane, AbdessamadIvanova, MiroslavaTrinidad Segovia, Juan E.Iyoda, EikiTripathy, Rajesh KumarIzadkhah, SalmanTripathy, SushantaJabbari, MehdiTrivellato, BarbaraJach, TomaszTroisi, AntonioJacobs, DonaldTrojan, JakubJacyna, MariannaTroshin, SergeyJaeger, GreggTroussas, ChristosJafari, GholamrezaTrovato, AntonioJafari, MohammadTrujillo, CarlosJagodziński, JacekTruong Nguyen, XuanJagtap, AmeyaTrushechkin, AntonJahnel, BenediktTsai, Chia-WeiJaiswal, AmareshTsai, Pin-JuJakimowicz, AleksanderTsallis, ConstantinoJakóbczyk, LechTsang, Yung PoJamalabadi, Mohammad Yaghoub AbdollahzadehTsanousa, AthinaJames, Lancelot F.Tsekouras, George E.James, Ryan GregoryTseng, Fan-ShuoJamnik, AndrejTseng, PhilipJamróz, DariuszTsiatsikas, ZisisJamshed, Muhammad AliTsihrintzis, George A.Jamshidi, MohammadTsiligkaridis, JohnJan, Chyan-LongTsionas, Mike G.Jañez-Martino, FranciscoTsitsas, NikolaosJanutka, AndrzejTsochev, GeorgiJarzynska, MariaTsoi, StephenJaskiewicz, MarekTsolis, ArisJaúregui, RocíoTsuneda, AkioJavarone, Marco AlbertoTsurkov, Vladimir IvanovichJaveed, ShumailaTsurutani, Bruce T.Javier, Hoyos-LeyvaTsuruyama, TatsuakiJędrzejewski, ArkadiuszTu, Chang-ShuJeon, Jun-CheolTu, Shu-FenJepps, OwenTu, Yu-JuJerbi, HoussemTuczyński, KrystianJessa, MieczyslawTudosa, IoanJevtic, MarijaTummala, SudhakarJha, PankajTumosa, NinaJiang, ChaozheTusset, Angelo MarceloJiang, JiahuanTwaróg, BogusławJiang, LiTyler, ChristopherJiang, MingTyrcha, JoannaJiang, NanUchida, SatoshiJiao, LianmengUddin, JiaJiménez, FernandoUeltzhoeffer, KaiJiménez-López, José DomingoUl Muram, FaizJin, BaisuoUlacha, GrzegorzJin, ChaoUlicny, MatejJin, JiasenUllah, NasimJing, NaihuanUllah, WaseemJing, YunUr Rehman, KhalilJipa, AlexandruUrban, FrankJiwari, RamUrban, JanJizba, PetrUrzua, AlejandroJo, KyuriUsacheva, TatianaJo, Soo-HoVacchini, BassanoJog, VarunVakili-Nezhaad, G. RezaJoglekar, YogeshVakulya, GergelyJohn, MajnuVala, JiriJones, Karl O.Valackienė, AstaJose, MarcoValdés-Hernández, AndreaJoseph, Sijo K.Valente, DanielJoshi, ChaitanyaValeriani, ChantalJoshi, Yogesh M.Valido, Antonio A.Jost, JuergenValkai, SándorJost, LouVallianatos, FilipposJuan, Justie Su-TzuValls, AidaJuel, Bjørn E.Valtierra-Rodriguez, MartinJuhar, JozefValverde-Albacete, Francisco J.Juhasz, ZoltanVan Andel, StevenJurić, TajronVan Enter, AernoutKaczmarczyk, Tomasz ZygmuntVan Isacker, PietKaden, MarikaVancea, Ion VasileKagalovsky, VictorVandevelde, LievenKainen, PaulVanetik, NataliaKajantie, KeijoVarando, GherardoKalaga, Joanna K.Vardasca, RicardoKalinin, SergeiVardi, AmichayKalinina, OlgaVarejão, Flávio MiguelKalkan, KaanVarga, DomonkosKalloniatis, ChristosVarga, Lajos KárolyKamps, UdoVargas-Solar, GenovevaKang, Dae-KiVarotsos, Panayiotis AntoniouKaniadakis, GiorgioVásárhelyi, JózsefKantartzis, Nikolaos V.Vashishtha, GovindKanter, Gregory S.Vattay, GáborKanukurthi, BhavanaVavoulis, Dimitrios V.Kaparulin, Dmitry S.Vaz de Melo, Pedro O. S.Kaplan, KaplanVázquez Polo, Francisco JoséKarafyllidis, Ioannis G.Vázquez, LuisKarakasidis, TheodorosVega-Oliveros, Didier AugustoKarakatsanis, LeonidasVeiga, Manuel FernándezKarakurt, Asım SinanVelázquez, RamiroKaramitsos, IoannisVelazquez-Perez, Jesus E.Karawia, Abdel Rahman A.Veloz, TomasKarimi, DanialVemparala, Manoj RohitKarimov, TimurVencalek, OndrejKarlos, StamatisVenegas-Martínez, FranciscoKarminsky, Alexander M.Ver Steeg, GregKarpat, FatihVerbelen, TimKarpat, GöktuğVerde, FrancescoKarthikeyan, KulandhaivelVerdière, NathalieKarydis, IoannisVerdoolaege, GeertKaryotis, VasileiosVergara, Jorge R.Kasamatsu, KenichiVergara, José DavidKasana, Singara SinghVerlekar, TanmayKashevnik, AlexeyVersaci, MarioKatkovnik, VladimirVerstraelen, WouterKaul, Sanjit KrishnanVertan, ConstantinKaur, ManjitVertse, TamasKavaliauskas, ŽydrunasVesnin, Artem M.Kavikumar, RamasamyVialetto, GiulioKavousanakis, MihalisVianna, Reinaldo OliveiraKawai, YujiVidal-Tomás, DavidKawakata, HironoriVideira, Romeu A.Kay, Jim W.Vidiella-Barrranco, AntonioKazak, JanViechtbauer, WolfgangKazakovtsev, LevViegas, EduardoKazanskiy, NikolayVieira, Salomé I. C.Kedem, YaronVieru, DumitruKelemenová, TatianaVîlcu, Gabriel-EduardKempa, WojciechVillafaina, SantosKenyeres, MartinVillani, MarcoKeriven, NicolasVillar, Paula I.Keser, TomislavVillas Boas, Celso J.Keshmiri, SoheilVillegas, PabloKetov, Sergei V.Vinko, DavorKetzmerick, RolandVinko, TamasKeziou, AmorVinoth Kumar, VenkatesanKhaled, AmeurVinte, ClaudiuKhalid, SalmanViolos, JohnKhalifa, AmalVirgo, NathanielKhan, Abdul SamadVisa, AureliaKhan, FirozViscito, LucaKhan, Muhammad HassanVisser, ManusKhan, WasiqViswanatha Sharma, KoradaKharchenko, VyacheslavVitanov, Nikolay K.Khare, NeeluVladyko, AndreiKhayati, MouradVlaj, DamjanKhlopov, MaximVogiatzis, ChrysafisKhodadadi, HamedVogl, ClausKhorshidi, Hadi AkbarzadehVogl, TobiasKhrennikov, AndreiVohryzek, JakubKhrennikova, PolinaVoicikas, AleksandrasKhvedelidze, ArsenVolodin, Alexander M.Kiebel, StefanVolpert, VitalyKielanowski, PiotrVon Tycowicz, ChristophKieu, ChanhVopson, Melvin M.Kilcik, AliVrba, JanKim, Chang SubVulkov, Lubin G.Kim, Chang-EunVysotska, VictoriaKim, Dong KyooWächtler, Christopher W.Kim, Dong-HeeWadayama, TadashiKim, Dong-KyuWadhawan, YatinKim, Eui SooWalek, BogdanKim, Eun-jinWales, DavidKim, Hee-YoungWalid, AichKim, IlkiWallace, ManolisKim, Jeong TaeWallot, SebastianKim, Jong HyoWalsh, ShaneKim, Jun MoWan, KaiKim, JunseokWan, ShaohuaKim, KyuseokWan, ShupingKim, MoonseongWang, Annie XuKim, Sang-JoonWang, BuyuKim, SooyoungWang, ChaohuiKinateder, HaraldWang, Chih-HungKing, Jean-RémiWang, ChuanKirchner, EckhardWang, ChunhuaKirillov, Alexander A.Wang, Danny J. J.Kirkley, AlecWang, DiKirmani, Syed N. U. A.Wang, Gang-JinKishore, RatnaWang, HaiKisielewicz, TomaszWang, HaoKiss, GaborWang, HongyiKizek, JánWang, KunKjelstrup, SigneWang, LinKlages, RainerWang, Lin-ShuKlampanos, Iraklis AngelosWang, NingKlančnik, GregaWang, Pi-ChungKlein, BrennanWang, PingKlein, LouisWang, QingKliber, PawełWang, ShugangKlika, VaclavWang, TaoKlimenko, AlexanderWang, TianboKlimov, AndreiWang, WeizhuoKluza, Paweł ArturWang, Wen-GeKłysz, SylwesterWang, WilliamKlyuev, Roman V.Wang, XikuiKnypiński, ŁukaszWang, XizhaoKo, HaneulWang, XuKo, TaehoonWang, YangKocharyan, Armen A.Wang, Yu-ChengKocherlakota, PrashantWaqas, HassanKoehler, MatthewWedemann, Roseli S.Koenig, Reto E.Wei, Hua-LiangKogias, DimitrisWei, ShuangqingKogut, IuriiWeichbroth, PawełKohler, SigmundWeidenmüller, Hans A.Koike, YutaWeigert, StefanKolchinsky, ArtemyWeinert, FriedelKolev, MikhailWeinfurter, HaraldKoley, SomnathWeiss, ChristianKolokoltsov, VassiliWeitschek, EmanuelKomljenovic, DraganWen, GuangruiKonda Gokuldoss, PrashanthWen, Kun-LiKondrashuk, IgorWen, XuedaKonečný, JaromírWeng, Chien-ErhKönig, JürgenWeng, Jian-JiaKononovicius, AleksejusWeron, TomaszKonstantinides, DimitriosWetzel, HannaKoprinkova-Hristova, PetiaWhitaker, AndrewKorbel, JanWhite, BrandieKořínek, TomášWibig, TadeuszKorotaev, SergeyWichert, AndreasKoshimura, MiyukiWięckowski, JakubKosloff, RonnieWieczorek, AleksanderKostic, MilivojeWielgosz, MaciejKotecha, KetanWiggins, BrandonKotsiantis, SotirisWijayanto, Arie WahyuKottaridi, ConstantinaWiktor, EjsmontKottke, DanielWilemski, GeraldKottursamy, KottilingamWilson, Stephen G.Kou, JiaqingWimsatt, William C.Kounchev, RoumenWinter, JohannesKountouris, MariosWio, OracioKoutmos, DimitriosWitanowski, ŁukaszKováč, ViliamWitczak, PiotrKováčová, MariaWitlox, FrankKovács, RóbertWłodarczyk, ZbigniewKovtanyuk, AndreyWohlschläger, AfraKowalewska-Kudłaszyk, AnnaWojciechowski, Krzysztof WitoldKozak, JanWollstadt, PatriciaKozin, ValeriiWongthanavasu, SartraKozioł, KarolWoo, JaeKozitsin, IvanWoźniak, MarcinKozłowski, EdwardWright, CoryKrak, YuriWright, StevenKrasnytska, MarianaWróbel, JoannaKrastev, VesselinWu, ChengyueKrasteva, VesselaWu, Hsien-TsaiKrastins, IvarsWu, HuamingKrause, AndreasWu, HuihuiKravchenko, Oleg V.Wu, Ja-LingKrim, HamidWu, JingKrinkin, KirillWu, JinranKrivovichev, Gerasim VladimirovichWu, Jong-WuuKröker, IljaWu, Jui-YuKrotov, DenisWu, JunKrygier, JaroslawWu, Pei-IngKrylov, GlebWu, Shin-TsonKuanar, ShibaWu, Shun-ChiKucherov, NikolayWu, Tsu-YangKudelcik, JozefWu, WenboKudryashov, BorisWu, YuchunKuehn, VolkerWu, ZhibinKuikka, VesaWübben, DirkKukal, JaromirXi, JianqiKułakowski, KrzysztofXia, FeiKulicki, PiotrXia, RongxinKumar, ApurvXiao, FuyuanKumar, AshutoshXiao, GuobaoKumar, AshwaniXiao, LiangliangKumar, NavinXiaojun, ChangKumar, SatishXie, YiqunKumar, VirendraXie, YunkunKumara, IndikaXiong, GuojiangKumbhakar, ManotoshXiong, NaixueKume, KensukeXu, ChenchuKundu, SrilenaXu, GuoxiaKuperman, MarceloXu, JunKuppusamy, VishnupriyaXu, NuKuri-Morales, Ángel FernandoXu, TanKůrková, VeraXu, YonghaoKurosaki, YuzuruYaguchi, TakaharuKurz, DariuszYakovyna, VitaliyKurzynsky, MichalYamagiwa, ShinichiKuś, MarekYamano, TakuyaKushner, TaisaYan, BinKuszewski, HubertYan, JiaKuti, JózsefYan, LeileiKuwalek, PiotrYan, QifaKuzu, Ridvan SalihYan, RuiKvalseth, TaraldYan, ZhengKvasov, Dmitri E.Yan, ZiqiKvet, MichalYanagisawa, DaichiKwek, Leong ChuanYanagisawa, HideyoshiKwok, Sze ChaiYang, CeKwon, Oh-MinYang, Chun-HaoKyaw, Thi HaYang, Chun-WeiKyriakopoulos, Grigorios L.Yang, Da-MingKyriakos, YakinthosYang, JanghoKyriazanos, DimitrisYang, JanghoonLa Cour, Brian R.Yang, LiangLabeur, Robert JanYang, MinoLabovsky, Alexander E.Yang, ShizhongŁadyżyńska-Kozdraś, EdytaYang, SonggeLafata, PavelYang, Yeon-MoLaguna, HumbertoYang, ZhenzhenLahmiri, SalimYankov, Metodi PlamenovLai, QiangYanofsky, Noson S.Laib, MohamedYao, QiweiLakshminarayan, ArulYao, YulingLamberti, Pedro WalterYasmin, HumairaLambert-Mogiliansky, ArianeYassaee, Mohammad HosseinLampart, PiotrYazdi, MohammadLamura, AntonioYazgan, ErdemLandajo, ManuelYe, NengLandsman, KlaasYe, ZhuolinLapidoth, AmosYeh, Bih ChyunLapuerta, María VictoriaYeh, Sheng-LihLapusan, CiprianYeh, Wei-ChangLara Molina, Fabian AndresYera Toledo, RacielLarralde, HernanYilmaz, MuhittinLarsen, LaurelYin, HanLasarzik, RobertYin, ShenLasoń, ArturYin, ShoulinLászló, KovácsYin, ZhengLatosh, BorisYin, ZhenyuanLau, Francis C. M.Yokose, SatoshiLau, John W.Youssef Moussa, Miled HassanLaurini, Márcio PolettiYu, Chang-ShuiLavanga, MarioYu, JingLavric, AlexandruYu, JinzhuLawniczak, MichalYu, ShujianLawrence, WalterYu, Simon C.Lázár, Zsolt IosifYu, TingLazaridis, ChristosYu, YangLe, Hoang D.Yuan, MengLeccadito, ArturoYuan, RuoshiLeclaire, SébastienYuan, XiaoLedziński, DamianYuan, ZhiliangLee, ChaohongYuji, IkedaLee, Ching-MinYukalov, Vyacheslav I.Lee, ChongdeukYun, Beong InLee, Hyung TaeYuste Delgado, Antonio JesúsLee, IlkeunZackay, BarakLee, Jae WooZafar, SohailLee, Je-InZafeiris, AnnaLee, JongwooZaffina, SalvatoreLee, Kyu-haengZaidi, AbdellatifLee, Ming-SuiZaitseva, ElenaLee, Ming-YueZajaček, MichalLee, Nam-YongZakharov, Anatoly Yu.Lee, Ray-KuangZambrano-Serrano, ErnestoLee, Sang HoonZambrini, Jean ClaudeLee, Sang JunZanella, MattiaLee, Sang WookZanette, Damián H.Lee, Sang-GonZanin, MassimilianoLee, SeonahZanotti, GiuseppeLee, SeunghwanZárate, Laura RosalesLee, SeungyupZarowski, MarcinLee, Sung-HakZarrin, JavadLee, Su-YongZavala-Yoe, RicardoLee, Tian-FuZazzaro, GaetanoLee, WoojooZdravevski, EftimLee, Woong-HeeZdziebko, TomaszLee, YanliZeeshan, AhmadLefta, FarhanZeghid, MedienLegnani, WalterZeid, IbrahimLeibovici, Didier G.Zekker, IvarLeinonen, MarkusZelenov, EvgenyLeipus, RemigijusZelevinsky, VladimirLeister, WolfgangZelezny, MilosLeitão, JoãoZeng, PeiLeithardt, Valderi Reis QuietinhoZenil, HectorLeitner, David M.Zet, CristianLeiva, VíctorZetterling, Carl-MikaelLengvarský, PavolZhang, ChrisLentini, Carlos A. D.Zhang, HaochunLeón, AlejandroZhang, HengjieLeon, GenlyZhang, JieLéonardon, MathieuZhang, LimingLeoński, WiesławZhang, MinglongLepori, LucaZhang, Shao-WuLeporini, DinoZhang, WeicunLeporini, RobertoZhang, Wen-RanLerma, ClaudiaZhang, XingchenLerner, PeterZhang, XuanLeszczyńska-Sejda, KatarzynaZhang, YanpengLeszczynski, JuliuszZhang, YonghaoLeung Patrick, Hui ChiZhang, YuanLevashenko, VitalyZhang, YugangLevin, MichaelZhang, YunchangLevina, AllaZhang, YunquanLevine, Zachary H.Zhang, ZhanjunLewandowsky, JanZhang, ZhedongLexi, XuZhang, ZhilangLey, ChristopheZhao, HongxiaLeyvraz, FrançoisZhao, LongfengLi, BingzhaoZhao, PeihaiLi, ChenZhao, TianLi, GuanchenZhao, YangLi, HaipengZhao, YunpengLi, HaitaoZhdanov, Vladimir P.Li, HuiZheng, ShenggenLi, JiaZheng, XinLi, JiakaiZheng, Zhu-JunLi, JiangZhokh, AlexeyLi, JianxinZhong, ChengwenLi, JingZhou, BoLi, JiyouZhou, FelixLi, JunZhou, Hai-JunLi, LeiZhou, HuabinLi, LinZhou, Jia-JiaLi, MengyaoZhou, LeiLi, MichaelZhou, QingguoLi, NaZhou, WeiLi, QiongZhou, XujuanLi, ShuaiZhou, YulunLi, ShuangyangZhu, ChangboLi, Tao E.Zhu, FukangLi, ThomasZhu, KeLi, TianchengZhu, QuanxinLi, WeipingZhu, YeyingLi, XinlingZidikheri, Meelis J.Li, YangZiemkiewicz, DavidLi, YaoxiangZikria, Yousaf BinLi, YongliZimmerman, WilliamLi, YongmingZinovyev, AndreiLi, YongqinZiolkowski, PatrykLi, ZhixiongZivieri, RobertoLi, ZuxingZlatev, ZlatinLiang, ShidongZlotnik, AlexanderLiang, XiangsanZoia, Maria GraziaLiang, YouZolek, NorbertLiao, FeixiongZollner, StefanLiao, XiaofengZolnai, ZsoltLiesegang, MoritzZorzi, MattiaLigęza, PawełZrelli, AmiraLikozar, BlažZubel, KrzysztofLima, Danielli Araújo



